# Fabrication of Defect-Free P84^®^ Polyimide Hollow Fiber for Gas Separation: Pathway to Formation of Optimized Structure

**DOI:** 10.3390/membranes10010004

**Published:** 2019-12-25

**Authors:** Miren Etxeberria-Benavides, Oguz Karvan, Freek Kapteijn, Jorge Gascon, Oana David

**Affiliations:** 1 TECNALIA, Parque Tecnológico de San Sebastián, Mikeletegi Pasealekua 2, 20009 Donostia-San Sebastián, Spain; o.karvan@utwente.nl; 2 Catalysis Engineering, Chemical Engineering Department, Delft University of Technology, Van der Maasweg 9, 2629 HZ Delft, The Netherlands; F.Kapteijn@tudelft.nl (F.K.); jorge.gascon@kaust.edu.sa (J.G.); 3 European Membrane Institute Twente (EMI), Faculty of Science and Technology, University of Twente, P.O. Box 217, 7522NB AE Enschede, The Netherlands; 4 KAUST Catalysis Center, Advanced Catalytic Materials, King Abdullah University of Science and Technology, Thuwal 23955, Saudi Arabia

**Keywords:** CO_2_/N_2_ separation, hollow fiber spinning, ultrathin skin layer, defect-free fibers

## Abstract

The elimination of the additional defect healing post-treatment step in asymmetric hollow fiber manufacturing would result in a significant reduction in membrane production cost. However, obtaining integrally skinned polymeric asymmetric hollow fiber membranes with an ultrathin and defect-free selective layer is quite challenging. In this study, P84® asymmetric hollow fiber membranes with a highly thin (~56 nm) defect-free skin were successfully fabricated by fine tuning the dope composition and spinning parameters using volatile additive (tetrahydrofuran, THF) as key parameters. An extensive experimental and theoretical study of the influence of volatile THF addition on the solubility parameter of the N-methylpyrrolidone/THF solvent mixture was performed. Although THF itself is not a solvent for P84®, in a mixture with a good solvent for the polymer, like N-Methyl-2-pyrrolidone (NMP), it can be dissolved at high THF concentrations (NMP/THF ratio > 0.52). The as-spun fibers had a reproducible ideal CO_2_/N_2_ selectivity of 40, and a CO_2_ permeance of 23 GPU at 35 °C. The fiber production can be scaled-up with retention of the selectivity.

## 1. Introduction

In order to assess gas separation at large scale, membrane products need to be highly productive. For this reason, there are two features that commercial membranes generally meet. First, the membranes are asymmetric with a dense and thin (100–200 nm) top layer supported by a thicker porous sublayer. The actual membrane is the top layer whose thickness controls the productivity of the membrane. Second, most commercial membranes are processed in the form of a fiber (<500 µm) with a hollow interior, because hollow fibers can be densely packed at over 10,000 m^2^ membrane active area in 1 m^3^ module volume [1,2,3], ten times more than for flat sheet membranes in plate and frame packaging.

The manufacture of asymmetric hollow fiber gas separation membranes follows a dry jet spinning and consecutive wet quench process [3,4,5,6,7,8]. The fabrication of hollow fibers with an ultrathin and defect-free dense selective layer is essential, since permeance and selectivity of the membrane will be determined by the quality of the selective layer. However, the thinner the selective layer is, the greater the probability for the creation of defects [9,10,11]. Therefore, obtaining ultrathin and defect-free selective layer is quite challenging. Since Henis and Tripodi reported that defects in asymmetric membranes could be repaired with a thin coating layer of a highly permeable polymer such as silicone rubber [12], healing techniques have been widely used to seal the defects. However, the defect healing post-treatment implies an additional step in membrane manufacturing, resulting in an increased membrane production cost. Therefore, significant efforts have been made during the last decades in the development of defect-free as-spun hollow fiber membranes. 

One approach was to add a highly volatile additive in the dope to facilitate the skin formation. Following this approach, defect-free Matrimid® 5218 hollow fiber membranes have been developed by several authors [13,14]. An ultra-thin selective layer of around 100 nm was obtained by Clausi and Koros [13] using spinning dopes comprising THF as volatile solvent. Krol et al. [14] used another highly volatile additive, i.e., acetone (b.p. = 56 °C). Hollow fibers with an effective top layer thickness of 300–400 nm were produced by tuning the polymer and acetone concentration in the spinning dope. High-flux and almost defect-free asymmetric hollow fiber membranes consisting of a 50/50 wt% P84®/Matrimid-blend were prepared by Visser et al. [15]. The volatile additive acetone was also used in the spinning dope to promote the formation of a skin layer of 43–73 nm. Asymmetric Torlon® polyamide-imide hollow fiber membranes with a defect-free selective skin layer of 410 nm were formed by Kosuri and Koros [16] by adding THF in the spinning dope. 

Defect-free as-spun Torlon® hollow fibers were successfully produced by Peng et al. [17] from a simple polymer/solvent spinning dope, resulting in ultra-thin dense layers of around 54 nm. Variation of the spinneret dimension and the take-up rate they managed to have a proper control of shear-induced and elongation-induced polymer chain orientation during the spinning process.

Polyimides are among the most interesting polymeric materials for gas separation applications due to their good trade-off between perm-selectivity and permeability, high thermal and chemical stability, combined with high mechanical strength, long durability and their suitability to prepare asymmetric structures [18]. P84®, a co-polyimide of 3,3´4,4´-benzophenone tetracarboxylic dianhydride and 80% methylphenylene-diamine + 20% methylene diamine or poly(BTDA-TDI/MDI), has been used for several applications such as organic solvent nanofiltration [19,20,21,22,23,24,25,26], pervaporation [27,28,29,30,31] and gas separation [15,32,33,34,35,36], alone or in a mixture with other polymers. 

Pure P84® hollow fiber membranes have been developed for gas separation by several authors [32,36,37,38]. Barsema et al. prepared highly selective P84® hollow fiber membranes and gas permeance, selectivity, and plasticization behaviour of pure and mixed was compared with dense flat sheet membranes. The reported CO_2_/N_2_, O_2_/N_2_ and He/N_2_ P84® selectivity values are among the highest in the literature [32]. In another publication, chemically cross-linked asymmetric P84® co-polyimide hollow fiber membranes with enhanced separation performance were fabricated by Choi et al. [36] using a dry-wet spinning process with an in-line cross linking step. An increased H_2_/CO_2_ selectivity and mechanical resistance was observed for the crosslinked hollow fiber membranes. In all these studies, however, P84® hollow fibers exhibit the intrinsic polymer selectivity for gas separation only after defect healing by silicone rubber coating.

Therefore, this work is focused on developing defect-free as-spun ultrathin P84® asymmetric hollow fiber membranes that do not require a silicone rubber coating post-treatment step. For this purpose, the approach reported in literature of using a dope solution comprising volatile and non-volatile solvents was followed. THF, one of the most studied volatile solvents for gas separation hollow fiber preparation, was selected as the volatile component in view of the positive influence on hollow fiber formation of other polyimides. The influence of volatile THF addition on the solubility parameter was extensively investigated, followed by spinning optimization through fine tuning of the dope composition and spinning parameters. The as-spun P84® asymmetric hollow fiber membranes were characterized by their performance in CO_2_ and N_2_ permeation.

## 2. Materials and Methods

### 2.1. Materials

P84® (BTDA-TDI/MDI) co-polyimide was supplied by HP Polymer GmbH. Anhydrous N-methylpyrrolidone (NMP, 99.5% purity) and tetrahydrofuran (THF, 99.5% purity) were purchased from Sigma-Aldrich (Madrid, Spain). Anhydrous ethanol (EtOH, ≥99.8% purity) was purchased from Prolabo. Hexane and methanol were purchased from Fisher Scientific (Madrid, Spain). All materials were used without further purification. 

### 2.2. Solubility Parameter Calculation 

The solubility parameter expresses the nature and magnitude of interaction forces working between polymers and solvents. The group contribution method was used to calculate the overall value of the solubility parameter [39]:

(1) $$\delta =\sqrt{\delta_{d}^{2}+\delta_{p}^{2}+\delta_{h}^{2}}$$

where *δ_d_*, *δ_p_* and *δ_h_* are the dispersive, polar and hydrogen bonding solubility parameters, respectively. The solubility parameter components may be calculated by the Hoftyzer and Van Krevelen method [40], using the following equations:

(2) $$\delta_{d}=\frac{\sum F_{di}}{V}\;;\;\;\;\;\;\;\;\;\;\;\;\;\;\delta_{p}=\frac{\sqrt{\sum F_{pi}^{2}}}{V}\;;\;\;\;\;\;\;\;\;\;\;\;\delta_{h}=\sqrt{\frac{\sum E_{hi}}{V}}$$

where *F_d_* is the dispersion component of the molar attraction constant, *F_p_* the polar component, *E_h_* the contribution of hydrogen bonding forces to the cohesive energy and *V* the molar volume.

The solubility of a polymer in an organic liquid can be determined by the total solubility parameter difference between polymer and solvent, i.e., the smaller the difference in the solubility parameter, the better the compatibility. It is calculated by the following equation, where each component of the Hansen solubility parameter of pure components (polymer, *P* and solvent, *S*) is taken into consideration:

(3) $$\Delta \delta_{P/S}=\sqrt{{\left(\delta_{d,P}-\delta_{d,S}\right)}^{2}+{\left(\delta_{p,P}-\delta_{p,S}\right)}^{2}+{\left(\delta_{h,P}-\delta_{h,S}\right)}^{2}}$$

### 2.3. Ternary Phase Diagram Determination

Ternary phase diagrams were determined via the cloud point technique at room temperature. Dopes containing different non-solvent (ethanol) concentrations were prepared for different polymer concentrations ranging from 15 to 30 wt%. The cloud point was assigned to the non-solvent concentration where the dope changes from one phase into two phases, i.e., when the solution become turbid for more than 5 min. The binodal line is formed joining the cloud points of different polymer concentrations. Ternary diagrams for two polymer/solvent/non-solvent systems (P84®/NMP/EtOH and P84®/NMP+THF/EtOH) were determined and the influence of the incorporation of THF was studied. Ternary diagrams for different NMP/THF ratios were determined. The mixture of NMP and THF is considered as an “effective solvent” to draw the ternary diagram.

### 2.4. Preparation of P84® Hollow Fiber Membranes

The fabrication of P84® hollow fiber membranes was based on a process of dry jet spinning followed by a wet quench [3,4,5,6,7,8]. The hollow fibers preparation procedure has been described in detail elsewhere [41]. A spinneret with the following dimensions was used: 630 µm inner diameter and 1200 µm outer diameter. After spinning, the fibers were solvent exchanged in a methanol bath for 30 min (3 times) followed by a hexane bath for 30 min (3 times) and dried at 70 °C overnight. Fibers were kept overnight in a vacuum oven at 100 °C to completely remove residual solvent.

### 2.5. Hollow Fiber Membrane Characterization

The surface and cross-section morphology of the hollow fiber membranes were characterized by scanning electron microscopy (SEM) (Quanta 250 ESEM, FEI, The Netherlands). Membrane samples were freeze-fractured after immersion in liquid nitrogen and subsequently coated with gold/palladium to analyse cross sections of the samples.

Hollow fiber membrane modules were prepared and tested using an experimental set-up, based on constant pressure technique [7] described in detail elsewhere [41]. Pure N_2_ and CO_2_ gas permeation experiments were carried out at a transmembrane pressure of 7 bar at 35 °C. Gas was fed from the lumen side of the fiber and permeate was collected from the shell side in a dead end, cross-flow configuration. The permeance for gas *i* was calculated by the following equation:

(4) $$P_{i}=\frac{F_{i}}{\Delta p_{i}\cdot A}$$

where *P_i_* is the gas permeance in gas permeation units (1 GPU = 10^−6^ cm^3^ (STP) cm^−2^ s^−1^ cmHg^−1^), *F_i_* is the volumetric flow rate of component *i* (cm^3^ (STP)/s), Δ*p_i_* is the partial pressure difference of component *i* across the membrane (cmHg) and *A* is the effective membrane area (cm^2^). The effective membrane area was calculated using the average value for fiber diameter taken from the measurement of 3 fibers at each end of the module and the active membrane length on the feed side. 

The ideal selectivity α*_ij_* was calculated as the ratio of the permeance of the more permeable compound *i* to the permeance of the less permeable compound *j*:

(5) $$\alpha_{ij}=\frac{P_{i}}{P_{j}}$$

## 3. Results and Discussion

### 3.1. Polymer Dope Optimization

Optimization of polymer solution (dope) composition is a key to success for the formation of defect-free hollow fiber membranes. For this purpose, the dope usually has a complex composition as it contains polymer, solvents and additives (non-solvents) that are miscible with the solvent but not with the polymer. Regarding polymer concentration, one requirement to produce hollow fibers with minimum defects is a high polymer concentration in order to create significant chain entanglement during skin formation. Nevertheless, a too high polymer concentration is not desired because it creates a support layer with lower porosity and higher resistance to gas transport. Therefore, the optimum value is met when the dope has sufficient viscosity to allow extrusion of the polymer dope and take-up at a relevant speed without breaking (i.e., a spinnable dope). In a polymer solution, the viscosity increases slightly with polymer concentration, up to a point from where it begins to increase exponentially. This point is called the critical polymer concentration (c.p.c.) and the optimum polymer concentration in the dope is equal to or slightly above this point. The critical P84® concentration of 28 wt% was identified from the viscosity vs. polymer concentration curve for a P84®/NMP dope solution by Peng et al. [42]. A P84® concentration of 28 to 28.5 wt% was employed in our spinning experiments.

The other components of the dope and their concentration are important for a rapid formation of a defect-free outer skin layer during the nascent fiber’s residence time in the air gap. In that sense, Xu et al. presented the qualitative dope composition trajectories in the ternary phase diagram during a dry-jet/wet-quench spinning process and concluded that potentially useful dope composition should be in the one-phase region and close to the binodal line [43]. By the use of a non-solvent the dope solution composition gets closer to the binodal boundary, facilitating a faster phase separation. Due to the strong non-solvent character of water titration of the spinning dope close to the binodal becomes difficult and therefore EtOH is commonly selected as non-solvent. Also, as presented in the introduction section, the evaporation of volatile components in the air gap causes an increase in polymer concentration in the outermost region of the fiber so a dense skin can be formed.

#### 3.1.1. Solubility Parameter

**Figure 1:**
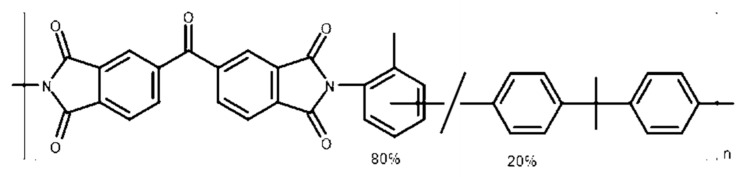
Chemical structure of P84® co-polyimide.

The influence of THF in the solubility of P84® in the solvent mixture NMP/THF was studied both theoretically and experimentally. Solubility parameter has been widely used to predict the solubility of polymers in various solvents. The solubility parameter of P84® co-polyimide was calculated by the group contribution method using Equations (1) and (2). The chemical structure of the polymer is shown in Figure 1 and the group contributions *F_di_*, *F_pi_* and *E_hi_* of the structural groups composing the co-polyimide are listed in Table 1. An overall value of the solubility parameter of 27.46 MPa^1/2^ was obtained for the P84® co-polyimide, in accordance with the value reported in literature for the same polymer [44]. 

The dispersive, polar and hydrogen bonding solubility parameter components, as well as the overall solubility parameter of components involved in the spinning dopes and phase inversion process, and the solubility parameter difference of P84® with the other components are presented in Table 2. 

**Figure 2:**
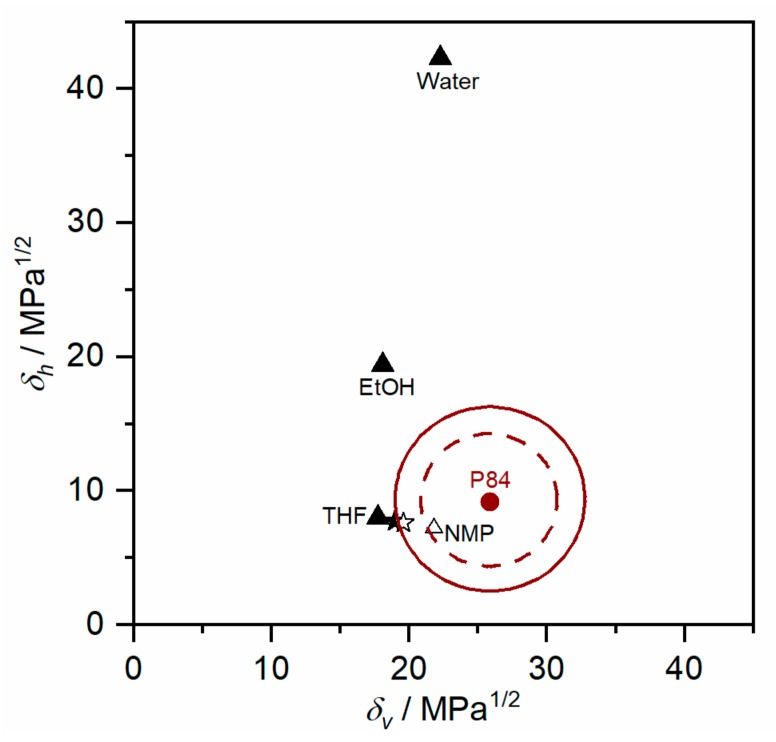
*δ_v_* − *δ_h_* diagram of P84® at room temperature. Solvents are represented by open symbols, and non-solvents by close symbols. Star symbols represent solvent mixture with a NMP/THF ratio of 1 (✰) and 0.52 (★). Theoretical (dashed line) and experimental (continuous line) “solubility circles” are represented.

The solubility parameter difference of P84® with the other components involved in the spinning dope increases in the order NMP (5.69) < THF (12.1) < EtOH (13.83). Thermodynamic considerations led to the conclusion that the effects of *δ_d_* and *δ_p_* over the Δ*δ_P/S_* are similar, while the effect of *δ_h_* is of a different nature. Accordingly, the parameter *δ_v_* = (*δ_d_* + *δ_p_* )^1/2^ was introduced, leading to *δ_v_* versus *δ_h_* diagrams [40]. In such diagrams, the interaction between a polymer and a number of solvents is graphically shown, and the “solubility circle” associated with the polymer is determined. The solubility region of the polymer is delimited by the circle, which usually has a radius of about 5δ-units. As a general rule, any liquid lying within the circle is a true solvent for the polymer, while the ones lying outside will act as non-solvents. The *δ_v_* − *δ_h_* diagram of P84® is presented in Figure 2, where solvent and non-solvent involved in the phase inversion process are included. NMP is situated within the theoretical “solubility circle” of P84® represented by a dashed line, and as consequence has been used as solvent for P84® membrane preparation in literature [32,42]. On the contrary, THF is out of the circle and therefore this solvent itself is not an appropriate solvent for P84®. However, a polymer could be dissolved in a mixture of two non-solvents or a mixture of a solvent and a non-solvent if the solubility parameter of the mixture is situated inside the circle.

**Figure 3:**
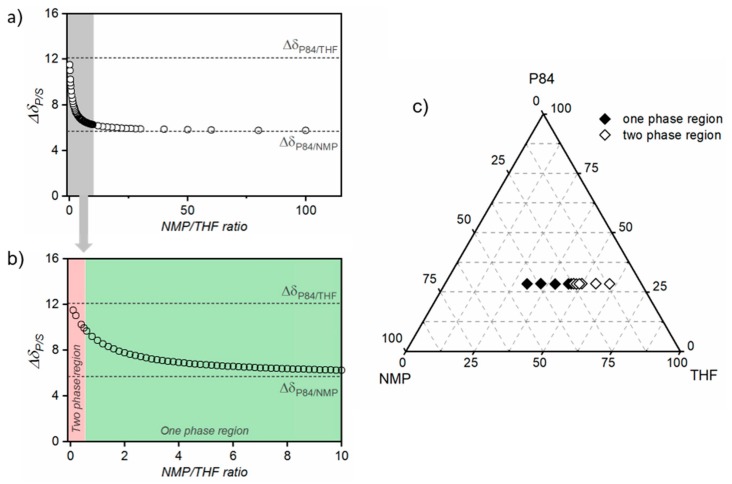
The difference in the solubility parameter of P84® and the solvent mixture (Δ*δ*_P84®/Smix_) as a function on NMP/THF ratio for (**a**) a NMP/THF ratio range of 0 to 120, and (**b**) a magnification of the 0 to 10 range. (**c**) Ternary phase diagram of P84®/NMP/THF system at room temperature. Close symbols represent solutions situated in the one phase region of the diagram and open symbols represent solutions situated in the two-phase region.

Therefore, the solubility parameter of the NMP/THF solvent mixture was calculated for different NMP/THF ratios. The difference in the solubility parameter of P84® and the solvent mixture (Δ*δ*_P84®/Smix_) is presented as a function on NMP/THF ratio in Figure 3a,b. As expected, Δ*δ*_P84®/Smix_ decreases as NMP/THF ratio increases (THF content decreases), showing an exponential decrease up to a NMP/THF ratio of 14 (from 12.1 to 6.1 MPa^1/2^, for pure THF and NMP/THF ratio of 14, respectively). Then, Δ*δ*_P84®/Smix_ continues decreasing more moderately up to 5.75 MPa^1/2^ (NMP/THF ratio of 100).

To experimentally determine the amount of THF that could be added to the spinning solution before it becomes unstable, dopes at different NMP/THF ratios (0.2 to 5.5) were prepared, at a constant P84® concentration of 28.5 wt%. As shown in Figure 3c, P84® solution becomes unstable at a THF concentration of ~47 wt%, which corresponds to a NMP/THF ratio of 0.52 (Δ*δ*_P84®/Smix_ value of 9.86 MPa^1/2^). Therefore, a NMP/THF ratio of 0.52 was defined as the boundary region for P84® dissolution and it was represented by a continuous line as the experimental “solubility circle” in Figure 2. In other words, P84® will solubilize in solvent mixtures with a NMP/THP ratio > 0.52. This demonstrated that even if THF itself is not an appropriate solvent for P84®, an NMP/THF mixture can be used to dissolve the polymer. The addition of THF weakens the solubilization capacity of the mixture. Nevertheless, a wide solubility window was identified for the NMP/THF solvent mixture, as reflected in green in Figure 3b.

#### 3.1.2. Ternary Phase Diagrams

**Figure 4:**
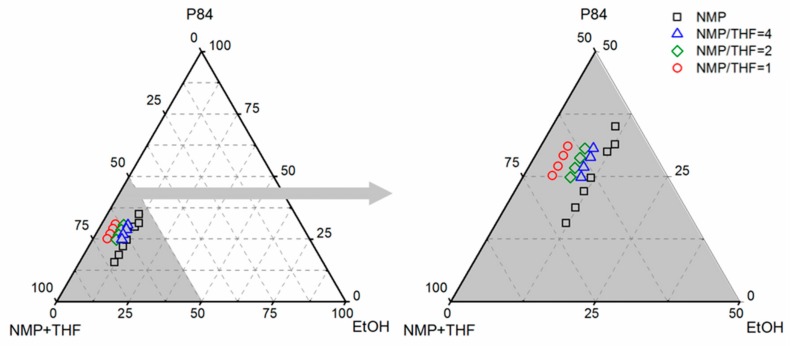
Ternary phase diagram of P84®/NMP/EtOH system (□) and P84®/(NMP/THF)/EtOH system at NMP/THF ratio of 4 (∆), 2 (◊) and 1(○) at room temperature.

Ternary phase diagrams for P84®/NMP/EtOH and P84®/NMP/THF/EtOH systems are presented in Figure 4. For P84®/NMP dope solution ~12 wt% ethanol is required to cause phase separation. As THF is incorporated in the system the binodal line is displaced closer to the polymer-solvent axis. For the P84®/NMP/THF dope solution with an NMP/THF ratio of 4, around 9 wt% of ethanol is required to cause phase separation. A further displacement of the binodal curve is observed as the THF concentration in the system increases, ~8 wt% and ~5 wt% ethanol is required to cause phase separation for a dope solution with an NMP/THF ratio of 2 and 1, respectively. This is in accordance with the solubility parameter study presented in Section 3.1. The dope solution becomes more unstable as the THF amount increases (Δ*δ*_P84®/Smix_ increases) and thus it can accommodate less non-solvent. 

### 3.2. P84® Hollow Fiber Spinning Process Optimization

Several spinning sessions were performed (D1–D5), the studied spinning parameter range, as well as separation performance of hollow fibers of each spinning sessions are presented in Table 3. For each spinning session a dope composition was established, and the spinning conditions were varied in order to find the optimal combination of spinning parameters for achieving the best performance for gas permeation. As-spun fiber separation performance was evaluated. These results are compared with gas permeation through a 20 µm thick dense flat film of P84® at similar operational conditions (1.28 Barrer CO_2_; 36.6 CO_2_/N_2_ selectivity at 35 °C) [32]. Asymmetric membranes are defined to be “defect-free” if the ideal selectivity is greater than 80% of the intrinsic selectivity of dense films [45]. In the case of asymmetric P84® hollow fibers this value has been fixed at a CO_2_/N_2_ selectivity of ~29.3 at 35 °C.

#### 3.2.1. P84®/NMP/EtOH Systems

For the first two spinning sessions (D1 and D2), a simple solvent system similar to that reported by Barsema et al. [32] was employed. The same solvent (NMP) and P84® concentration was employed (28.5 wt%), but with the only difference of the incorporation of ethanol to the dope. Ethanol was added into the dopes to be close to the binodal line (7 wt% and 9 wt% of ethanol to D1 and D2, respectively). The high permeance and lack of selectivity of as-spun fibers (>780 GPU of CO_2_ and ~1 CO_2_/N_2_ selectivity) denoted that highly defective fibers were obtained, even when the ethanol concentration was increased for D2 to be even closer to the binodal curve. 

#### 3.2.2. P84®/NMP/THF/EtOH Systems

**Figure 5:**
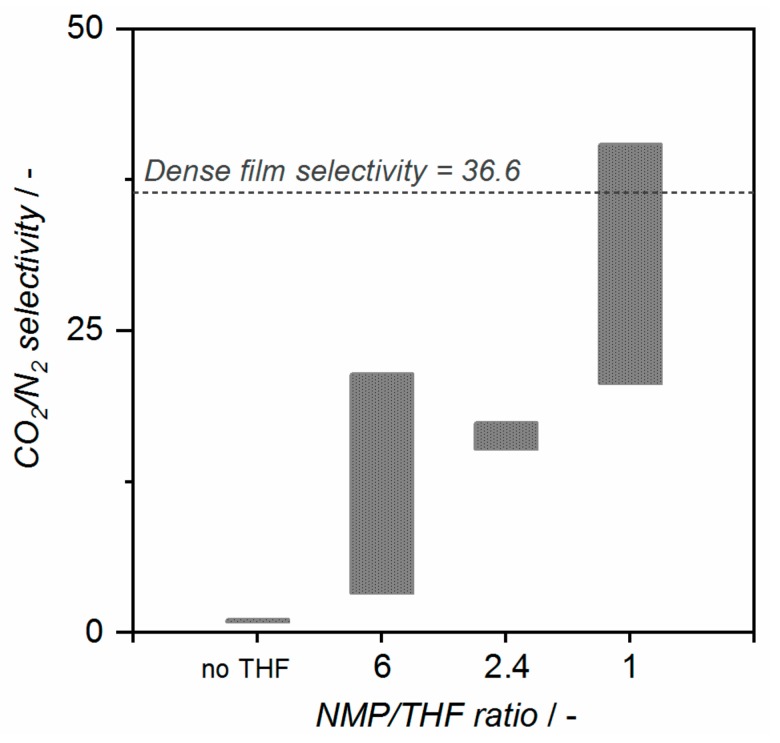
Ideal CO_2_/N_2_ selectivity range obtained at 35 °C for fibers spun from dopes D1 and D2 (no THF), D3 (NMP/THF ratio 6), D4 (ratio 2.4) and D5 (ratio 1).

The addition of THF to the spinning dope results on the obtention of less defective fibers. Figure 5 shows that an increase in ideal CO_2_/N_2_ selectivity is observed by decreasing the NMP/THF ratio (THF content increases). Dope D3, with an NMP/THF ratio of 6, gives fibers with CO_2_ permeances lower than 193 GPU and CO_2_/N_2_ selectivity ranging from 3.28 to 21.4. A further increase in THF content in D4 (NMP/THF ratio of 2.4) promotes the formation of a tighter skin layer and therefore, less permeable fibers (CO_2_ permeance < 35 GPU). However, the obtained selectivity value ranging from 15.2 to 17.4, almost half of the dense film selectivity value, denoted the presence of small defects in the selective layer of the fibers. The THF content was further increased to NMP/THF ratio of 1 for the D5 spinning session. Also, in order to accelerate phase separation LiNO_3_ was introduced in the system [43]. For all prepared fibers, an ideal CO_2_/N_2_ selectivity higher than 20 was obtained. Even more, a selectivity of 40.4 was obtained for one of the states, higher than the dense film selectivity values. This phenomenon has been reported before for 6FDA-based polymer spinning [46,47]. It was hypothesized, that it was due to the uniaxial orientation of polymer chains resulting from the high shear rate in the spinneret. This results in a tighter packing of the polymer chains, leading to an increase in selectivity over the unaligned state of polymer chains of the dense film. This demonstrates that as-spun fibers with a small number of defects or even defect-free fibers can be obtained by fine tuning of the dope composition and spinning parameters.

The performance of the four different fibers states obtained from dope D5 are analyzed in detail. Spinning conditions, separation performance and selective layer thickness of the four states of hollow fiber membranes are presented in Table 4. The selective layer thickness was estimated from the intrinsic CO_2_ permeability of the dense film and the permeance value of the asymmetric hollow fiber membrane. The influence of two spinning parameters was studied: The air gap height and the spinneret temperature.

**Figure 6:**
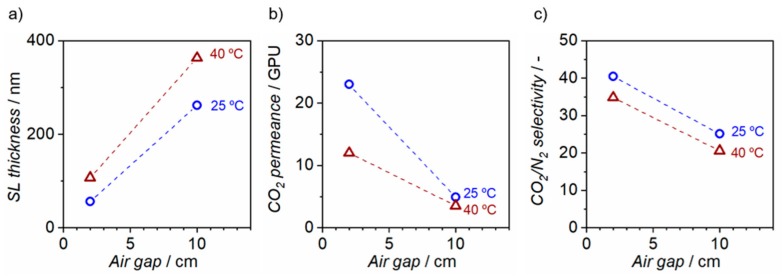
Air gap vs. (**a**) selective layer thickness, (**b**) CO_2_ permeance at 35 °C and (**c**) CO_2_/N_2_ selectivity for two spinneret temperatures (25 °C and 40 °C)

Selective layer thickness, CO_2_ permeance and ideal CO_2_/N_2_ selectivity as a function of the air gap for two spinneret temperatures (25 and 40 °C) are presented in Figure 6. The selective layer thickness is increased by the increase of the air gap (from 2 to 10 cm) for both spinneret temperatures, from 56 to 262 nm and from 107 to 363 nm for a spinneret temperature of 25 and 40 °C, respectively. Correspondingly, a decrease in CO_2_ permeance is observed by the increased air gap height. The dense selective layer is formed by the evaporation of the solvent in the air gap, mostly volatile THF. As the air gap height increases, the residence time of the fiber in the air gap increases, and hence, the evaporated solvent amount, increasing the polymer concentration at the outermost region of the fiber. Therefore, an increase in selective layer thickness and a decrease in gas permeance is expected by increasing the air gap.

Figure 6 also shows that an increased selective layer thickness and a decreased CO_2_ permeance is observed for the higher spinneret temperature. The increased spinneret temperature may induce a larger amount of evaporated solvent and therefore an increased selective layer thickness and hence a decreased CO_2_ permeance.

**Figure 7:**
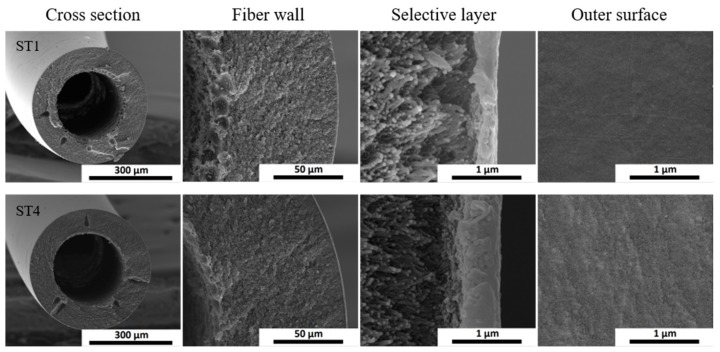
SEM images of the cross-section (overall fiber, fiber wall and the selective layer) and outer surface of hollow fibers from ST1 (air gap: 2 cm and spinneret temperature 25 °C) and ST4 (air gap: 10 cm and spinneret temperature 40 °C).

SEM images of the cross-section of the overall fiber, fiber wall and the selective layer as well as the outer surface of hollow fiber states prepared at the lowest air gap and spinneret temperature (ST-1) and highest air gap and spinneret temperature (ST-4) are presented in Figure 7. Both fibers present an outer diameter of ~430 µm and a similar sponge-like substructure. The trend observed in the estimation of the selective layer thickness from the CO_2_ permeance value (i.e., 56 nm for ST-1 and 363 nm for ST-4) is somehow confirmed by the SEM images, showing a thicker selective layer of ~500 nm for the latter. The selective layer thickness measured by SEM is higher than the one given by CO_2_ permeance due to bending of the outer layer produced during the freeze-fracturing of the sample in liquid nitrogen. Although a cleaner cut could improve the estimation of the selective layer thickness by SEM, in most cases would not be easy to identify the borderline between selective layer/transition layer/porous support. In addition, SEM analysis is local and did not give an average. Although the calculation of selective layer thickness from CO_2_ permeance value is also a simple estimation—it does not consider substrate resistance to the overall membrane resistance—it is useful for evaluating if the produced fibers have the thinnest achievable selective layer (i.e., ~100 nm) or if there is still room for optimization.

On the other hand, Figure 6c shows that the threshold selectivity value to consider P84® fibers as defect-free (CO_2_/N_2_ ~29.3) is attained only for the lowest air gap height of 2 cm for both spinneret temperatures. This result is in contradiction with the expected increase in selectivity with the increase in solvent evaporation rate at larger air gaps. We speculate that, during the residence time of the spun fiber in the air gap, an early phase separation on the outer surface of the fiber could be induced by water absorption from the ambient humidity. This may cause formation of small defects in the outer dense selective layer. A short air gap helps to minimize the effect of the water vapor within the air gap, and therefore, the number of defects. Nevertheless, the obtained selectivity value of 20–25 for the maximum air gap of 10 cm suggests that the defects could be easily healed by the conventional silicone rubber coatings.

The highest permeability and ideal selectivity were obtained for fibers spun with the smallest air gap (2 cm) and lowest spinneret temperature (25 °C). An ultra-thin selective layer of ~56 nm was obtained, almost ten times lower than the 500 nm thick selective layer obtained by Barsema et al. [32]. These hollow fiber membranes present an ideal CO_2_/N_2_ selectivity of 40.4, and the highest permeance reported in literature for P84® hollow fibers, 23 GPU of CO_2_ at 35 °C, against 2.2 GPU at 25 °C reported by Barsema et al.

**Figure 8:**
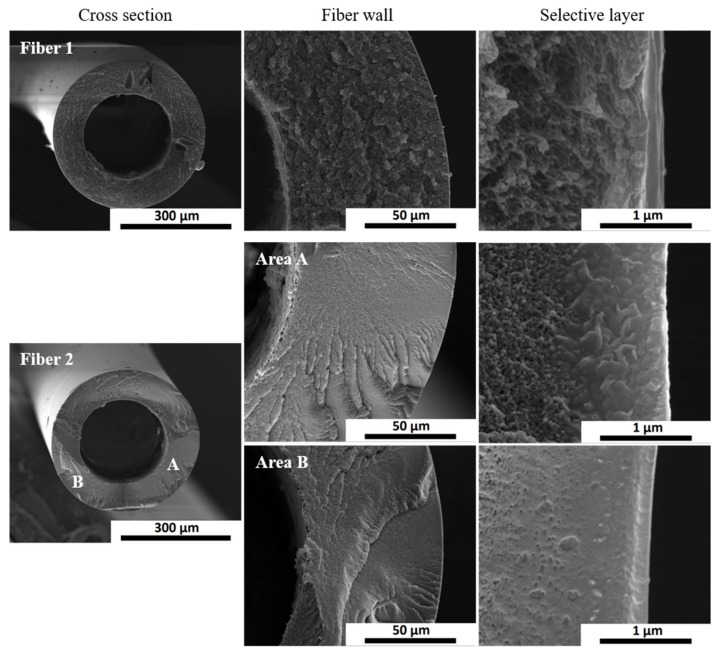
SEM images of the cross-section (overall fiber, fiber wall and the selective layer) of up-scaled D5_ST1 hollow fibers.

A new spinning process has been performed at a larger scale (~5000 meters of fibers) using the optimal spinning parameters from D5, ST1 with the difference that we used a flow of dry N_2_ in the air gap with the objective of eliminating humidity influence. Five modules containing 10 fibers each, taken at different production times have been characterized and the average value is provided in Table 5. For comparison purposes, the hollow fiber performance for the reference spinning process is included. The ideal selectivity was reproduced for the scaled up fibers, while the CO_2_ permeance was lowered. We assume there was a combination of factors that gave lower CO_2_ permeance, like faster evaporation of solvent induced by forced N_2_ flow in the air gap, or small variation in the other spinning parameters like room temperature (15 °C for spinning D5_ST1 versus 25 °C for up-scale D5_ST-1). SEM images of up-scaled fibers are presented in Figure 8. Two fibers were subject to SEM analysis and several areas of the selective layer were checked. A selective layer thickness of around 200 nm could be estimated from the clearest cut of fiber 2 area B, slightly higher than the one estimated from CO_2_ permeance value.

## 4. Conclusions

Using volatile THF in combination with non-volatile NMP allows the fabrication of defect-free ultra-thin (~56 nm) P84® asymmetric hollow fiber membranes with no need for defect healing post treatment. The elimination of an additional defect healing post-treatment step would result in a significant reduction in membrane production cost. Solubility parameter study demonstrated that even while THF itself is not an appropriate solvent for P84®, an NMP/THF mixture could be used to dissolve the polymer as long as the NMP/THF ratio is kept above 0.52. Defect-free as-spun P84® hollow fiber membranes fibers can be obtained by fine tuning of the dope composition and spinning parameters. The best results were obtained for hollow fibers membranes spun from a spinning dope containing a NMP/THF ratio of 1 and the smallest air gap and spinneret temperature studied (2 cm and 25 °C), resulting in a CO_2_ permeance improvement by a factor ten and an ideal CO_2_/N_2_ selectivity of up to 40. The spinning process is reproducible at a larger scale (~5000 m fibers).

## Figures and Tables

**Table 1:** Group contribution to P84® (BTDA-TDI/MDI) co-polyimide [40].

Functional Group	Numbers	*F_di_* (*J*^1/2^ m^3/2^ mol^−1^)	*F_pi_* (*J*^1/2^ m^3/2^ mol^−1^)	*E_hi_* (*J* mol^−1^)	*V_i_* (cm^3^ mol^−1^)
–CH_3_	0.8	420	0	0	21.9
–CH_2_	0.2	270	0	0	16.4
=CH–	10	200	0	0	13.5
=C–	9.2	70	0	0	0.0
–CO–	5	290	770	2000	13.6
–N=	2	20	800	5000	6.9

**Table 2:** Calculated solubility parameters of components involved in the spinning dopes and coagulation bath [39].

Component	*δ_d_* (MPa^1/2^)	*δ_p_* (MPa^1/2^)	*δ_h_* (MPa^1/2^)	*δ* (MPa^1/2^)	∆*δ_P/S_* (MPa^1/2^)
P84®	19.0	17.5	9.17	27.5	-
NMP	18.0	12.3	7.20	23.0	5.69
THF	16.8	5.7	8.00	19.5	12.1
EtOH	15.8	8.80	19.4	26.5	13.8
Water	15.5	16	42.3	47.8	33.4

**Table 3:** Spinning conditions and permeation performance for P84® hollow fiber membranes prepared from different dope compositions. Single gas permeation at 35 °C and 7 bar transmembrane pressure. The permeance data are values of two modules of ten fibers each.

**Spinning Parameters**	**Unit**	**D1**	**D2**	**D3**	**D4**	**D5**
Dope composition	wt% P84® wt% NMP wt% THF wt% EtOH	28.5 64.5 - 7	28.5 62.5 - 9	28.5 58.7 9.8 3	28 46.9 19.1 6	28.5 35.2 35.3 1 *
NMP/THF ratio	-	-	-	6	2.4	1
Dope/bore fluid flow rate	mL/h	180/60	180/60	180/60	180/60	210/80, 240/90
Bore fluid composition	NMP/H_2_O	87.5/12.5	93/7, 85/15	84.5/15.5	84.8/15.2	87/13
Spinneret temperature	°C	25	30–40	25–30	25–30	25–40
Air gap height	cm	5–15	5–15	5–15	5–10	2–10
Quench bath temperature	°C	25	25	25	25	25
Take up rate	m/min	20–30	20–25	20–30	20–30	20
**Membrane Performance**	**Unit**	**D1**	**D2**	**D3**	**D4**	**D5**
CO_2_ permeance	GPU	2269–1692	784–5692	79.9–193	28.2–34.9	3.53–23.0
Ideal CO_2_/N_2_ selectivity	-	0.98–1.08	0.87–1.09	3.28–21.4	15.2–17.4	20.6–40.4

* LiNO_3_.

**Table 4:** Spinning conditions and separation performance of the four states of hollow fiber membranes prepared from dope D5. Membrane performance for single gas permeation at 35 °C and 7 bar transmembrane pressure. The permeance data are average values of two modules of ten fibers each and error correspond to standard deviation.

**-**	**D5**	**Unit**	**ST-1**	**ST-2**	**ST-3**	**ST-4**
Spinning Parameters	Spinneret temperature	°C	25	25	40	40
	Air gap height	cm	2	10	2	10
Separation Performance	CO_2_ permeance	GPU	23.0 ± 1.8	4.9 ± 0.1	12.0 ± 1.5	3.5 ± 0.1
	Ideal CO_2_/N_2_ selectivity	-	40.4 ± 1.4	25.1 ± 0.4	34.8 ± 2.8	20.6 ± 0.25
	Selective layer thickness (calculated from permeance)	nm	56	262	107	363

Only varied spinning parameters are listed on the table, the rest of the parameters were kept constant: Dope composition (28.5 wt% P84®/35.2 wt% NMP/35.3 wt% THF/1 wt% LiNO_3_), bore fluid composition (87 wt%/13 wt% NMP/H_2_O), quench bath temperature (25 °C) and take up rate (20 m/min). Dope flow rate and bore flow rate were 210 mL/h and 80 mL/h, respectively, except for ST-4 where dope and bore fluid flow rate were increased to 240 mL/h and 90 mL/h to avoid pulsing phenomena observed at lower values.

**Table 5:** Separation performance of hollow fiber membranes prepared from dope D5 and spinning conditions ST-1 in two spinning sessions (reference and scaled-up). Separation performance for single gas permeation at 35 °C and 7 bar transmembrane pressure.

Separation Performance	Unit	D5_ST-1	Up-Scale D5_ST-1
CO_2_ permeance	GPU	23.0 ± 1.8	8.4 ± 1.7
Ideal CO_2_/N_2_ selectivity	-	40.4 ± 1.4	39.1 ± 1.0
Selective layer thickness (calculated from permeance)	nm	56	152

## References

[1] Wahab M.F.A., Ismail A.F., Shilton S.J. (2012). Studies on gas permeation performance of asymmetric polysulfone hollow fiber mixed matrix membranes using nanosized fumed silica as fillers. Sep. Purif. Technol..

[2] Husain S., Koros W.J. (2007). Mixed matrix hollow fiber membranes made with modified HSSZ-13 zeolite in polyetherimide polymer matrix for gas separation. J. Membr. Sci..

[3] Peng N., Widjojo N., Sukitpaneenit P., Teoh M.M., Lipscomb G.G., Chung T.-S., Lai J.-Y. (2012). Evolution of polymeric hollow fibers as sustainable technologies: Past, present, and future. Prog. Polym. Sci..

[4] Li Y., Chung T., Huang Z., Kulprathipanja S. (2006). Dual-layer polyethersulfone (PES)/BTDA-TDI/MDI co-polyimide (P84) hollow fiber membranes with a submicron PES–zeolite beta mixed matrix dense-selective layer for gas separation. J. Membr. Sci..

[5] Widjojo N., Chung T.-S., Kulprathipanja S. (2008). The fabrication of hollow fiber membranes with double-layer mixed-matrix materials for gas separation. J. Membr. Sci..

[6] David O., Gendel Y., Wessling M. (2014). Tubular macro-porous titanium membranes. J. Membr. Sci..

[7] David O.C., Gorri D., Nijmeijer K., Ortiz I., Urtiaga A. (2012). Hydrogen separation from multicomponent gas mixtures containing CO, N_2_ and CO_2_ using Matrimid® asymmetric hollow fiber membranes. J. Membr. Sci..

[8] Babu V.P., Kraftschik B.E., Koros W.J. (2018). Crosslinkable TEGMC asymmetric hollow fiber membranes for aggressive sour gas separations. J. Membr. Sci..

[9] Yampolskii Y. (2012). Polymeric Gas Separation Membranes. Macromolecules.

[10] Peng N., Chung T.-S., Lai J.-Y. (2009). The rheology of Torlon® solutions and its role in the formation of ultra-thin defect-free Torlon® hollow fiber membranes for gas separation. J. Membr. Sci..

[11] Pinnau I., Koros W.J. (1992). Gas-permeation properties of asymmetric polycarbonate, polyestercarbonate, and fluorinated polyimide membranes prepared by the generalized dry–wet phase inversion process. J. Appl. Polym. Sci..

[12] Henis J.M.S., Tripodi M.K. (1981). Composite hollow fiber membranes for gas separation: The resistance model approach. J. Membr. Sci..

[13] Clausi D.T., Koros W.J. (2000). Formation of defect-free polyimide hollow fiber membranes for gas separations. J. Membr. Sci..

[14] Krol J.J., Boerrigter M., Koops G.H. (2001). Polyimide hollow fiber gas separation membranes: Preparation and the suppression of plasticization in propane/propylene environments. J. Membr. Sci..

[15] Visser T. (2006). Mixed Gas Plasticization Phenomena in Asymmetric Membranes.

[16] Kosuri M.R., Koros W.J. (2008). Defect-free asymmetric hollow fiber membranes from Torlon^®^, a polyamide–imide polymer, for high-pressure CO_2_ separations. J. Membr. Sci..

[17] Peng N., Chung T.S. (2008). The effects of spinneret dimension and hollow fiber dimension on gas separation performance of ultra-thin defect-free Torlon ® hollow fiber membranes. J. Membr. Sci..

[18] Kase Y. (2008). Gas Separation by Polyimide Membranes. Adv. Membr. Technol. Appl..

[19] Ba C., Langer J., Economy J. (2009). Chemical modification of P84 copolyimide membranes by polyethylenimine for nanofiltration. J. Membr. Sci..

[20] See-Toh Y.H., Silva M., Livingston A. (2008). Controlling molecular weight cut-off curves for highly solvent stable organic solvent nanofiltration (OSN) membranes. J. Membr. Sci..

[21] See Toh Y.H., Lim F.W., Livingston A.G. (2007). Polymeric membranes for nanofiltration in polar aprotic solvents. J. Membr. Sci..

[22] Vandezande P., Gevers L.E.M., Vankelecom I.F.J. (2008). Solvent resistant nanofiltration: Separating on a molecular level. Chem. Soc. Rev..

[23] Han R., Xie Y., Ma X. (2019). Crosslinked P84 copolyimide/MXene mixed matrix membrane with excellent solvent resistance and permselectivity. Chin. J. Chem. Eng..

[24] Han R., Xie Y., Ma X., Teng D., Zhang S., Jian X. (2019). Preparation of poly(2,4,6-triaminopyrimidine-TMC)/P84 composite nanofiltration membrane with enhanced chlorine resistance and solvent resistance. J. Chem. Technol. Biotechnol..

[25] Davood Abadi Farahani M.H., Chung T.-S. (2018). Solvent resistant hollow fiber membranes comprising P84 polyimide and amine-functionalized carbon nanotubes with potential applications in pharmaceutical, food, and petrochemical industries. Chem. Eng. J..

[26] Dutczak S.M., Tanardi C.R., Kopeć K.K., Wessling M., Stamatialis D. (2012). “Chemistry in a spinneret” to fabricate hollow fibers for organic solvent filtration. Sep. Purif. Technol..

[27] Polotskaya G., Putintseva M., Pulyalina A., Gofman I., Toikka A. (2018). Impact of endometallofullerene on P84 copolyimide transport and thermomechanical properties. Polymers.

[28] Shi G.M., Wang Y., Chung T.-S. (2012). Dual-layer PBI/P84 hollow fibers for pervaporation dehydration of acetone. AIChE J..

[29] Liu R., Qiao X., Chung T.-S. (2005). The development of high performance P84 co-polyimide hollow fibers for pervaporation dehydration of isopropanol. Chem. Eng. Sci..

[30] Qiao X., Chung T.-S., Pramoda K.P. (2005). Fabrication and characterization of BTDA-TDI/MDI (P84) co-polyimide membranes for the pervaporation dehydration of isopropanol. J. Membr. Sci..

[31] Qiao X., Chung T.-S. (2005). Fundamental Characteristics of Sorption, Swelling, and Permeation of P84 Co-polyimide Membranes for Pervaporation Dehydration of Alcohols. Ind. Eng. Chem. Res..

[32] Barsema J.N., Kapantaidakis G.C., van der Vegt N.F.A., Koops G.H., Wessling M. (2003). Preparation and characterization of highly selective dense and hollow fiber asymmetric membranes based on BTDA-TDI/MDI co-polyimide. J. Membr. Sci..

[33] Visser T., Masetto N., Wessling M. (2007). Materials dependence of mixed gas plasticization behavior in asymmetric membranes. J. Membr. Sci..

[34] Omidvar M., Stafford C.M., Lin H. (2019). Thermally stable cross-linked P84 with superior membrane H_2_/CO_2_ separation properties at 100 °C. J. Membr. Sci..

[35] Guo A., Ban Y., Yang K., Yang W. (2018). Metal-organic framework-based mixed matrix membranes: Synergetic effect of adsorption and diffusion for CO_2_/CH_4_ separation. J. Membr. Sci..

[36] Choi S.-H., Jansen J.C., Tasselli F., Barbieri G., Drioli E. (2010). In-line formation of chemically cross-linked P84^®^ co-polyimide hollow fibre membranes for H_2_/CO_2_ separation. Sep. Purif. Technol..

[37] Favvas E.P., Papageorgiou S.K., Nolan J.W., Stefanopoulos K.L., Mitropoulos A.C. (2013). Effect of air gap on gas permeance/selectivity performance of BTDA-TDI/MDI copolyimide hollow fiber membranes. J. Appl. Polym. Sci..

[38] Sheng L., Ren J., Hua K., Li H., Feng Y., Deng M. (2019). The enhancement of mechanical properties of P84 hollow fiber membranes by thermally annealing below and above Tg. J. Membr. Sci..

[39] Hansen C.M., Press C. (2007). Hansen Solubility Parameters: A User’s Handbook.

[40] Van Krevelen D.W., te Nijenhuis K., Science E. (2009). Properties of Polymers: Their Correlation with Chemical Structure; Their Numerical Estimation and Prediction from Additive Group Contributions.

[41] Etxeberria-Benavides M., Johnson T., Cao S., Zornoza B., Coronas J., Sanchez-Lainez J., Sabetghadam A., Liu X., Andres-Garcia E., Kapteijn F. (2019). PBI mixed matrix hollow fiber membrane: Influence of ZIF-8 filler over H_2_/CO_2_ separation performance at high temperature and pressure. Sep. Purif. Technol..

[42] Peng N., Chung T.S., Wang K.Y. (2008). Macrovoid evolution and critical factors to form macrovoid-free hollow fiber membranes. J. Membr. Sci..

[43] Xu L., Zhang C., Rungta M., Qiu W., Liu J., Koros W.J. (2014). Formation of defect-free 6FDA-DAM asymmetric hollow fiber membranes for gas separations. J. Membr. Sci..

[44] Soroko I., Lopes M.P., Livingston A. (2011). The effect of membrane formation parameters on performance of polyimide membranes for organic solvent nanofiltration (OSN): Part A. Effect of polymer/solvent/non-solvent system choice. J. Membr. Sci..

[45] Pesek S.C., Koros W.J. (1993). Aqueous quenched asymmetric polysulfone membranes prepared by dry/wet phase separation. J. Membr. Sci..

[46] Chung T.-S., Lin W.-H., Vora R.H. (2000). The effect of shear rates on gas separation performance of 6FDA-durene polyimide hollow fibers. J. Membr. Sci..

[47] Lively R.P., Dose M.E., Xu L., Vaughn J.T., Johnson J.R., Thompson J.A., Zhang K., Lydon M.E., Lee J.-S., Liu L. (2012). A high-flux polyimide hollow fiber membrane to minimize footprint and energy penalty for CO2 recovery from flue gas. J. Membr. Sci..

